# Unlocking the World of Travel for Individuals With Intellectual and Developmental Disabilities

**DOI:** 10.7759/cureus.51846

**Published:** 2024-01-08

**Authors:** Alice Hamilton, Rafik Jacob

**Affiliations:** 1 Internal Medicine, University of Florida College of Medicine – Jacksonville, Jacksonville, USA

**Keywords:** behavioral issues, financial difficulties, pre-travel medical assessment, primary care physicians, challenges, travel, intellectual and developmental disabilities

## Abstract

This editorial explores the challenges faced by families with individuals with intellectual and developmental disabilities (IDD) when it comes to travel, drawing parallels with the difficulties experienced in attending routine doctor's appointments. The disruptions to routine, preparation of supplies, and the fear of unfamiliar environments often make travel seem like an unattainable dream for these families. Despite these challenges, some families showcase resilience and determination, managing to travel with their loved ones. The article emphasizes the inconsistency in experiences across families due to varying levels of cognition, adaptive functioning, financial means, and available support. It discusses the additional complications for families dealing with medical procedures, the scrutiny of strangers, and financial difficulties. The article suggests the crucial role of primary care physicians in facilitating travel for these families by performing pre-travel medical assessments, consulting social workers, and preparing comprehensive emergency plans. It proposes the need for collaboration between governments, the tourism industry, advocacy groups, and the community to address these challenges. Ultimately, the article advocates for the empowerment of families with IDD individuals to enjoy the world as tourists, with the support of their primary care providers.

## Editorial

Travel has the power to transform lives, but for families of individuals with intellectual and developmental disabilities (IDD), it often seems like an unattainable dream. In this article, we explore the challenges faced by these families and how to overcome them.

Put yourself in the shoes of a family with an individual with intellectual and developmental disabilities, trying to arrive at a doctor’s appointment in a timely manner. To begin with, this experience often disrupts a carefully and lovingly crafted routine that has been effective for the family. Additionally, it involves hours of preparation for necessary supplies, moving mobility equipment, loading into the car, unexpected stops during the journey, and ultimately, prolonged waiting in various office rooms that are often unable to accommodate them effectively. How do you think these challenges would impact your willingness to schedule future appointments?

Imagine having to manage all the tasks mentioned above, which includes a significant financial investment for the trip, all while being in an unfamiliar environment with people you've never met. This is the reality for families traveling with an individual with IDD. The fear of the unknown and the potential challenges that may arise can be daunting enough to discourage anyone from embarking on such a journey. However, there are remarkable families that have demonstrated incredible resilience and determination by making extraordinary efforts to travel with their loved ones who have IDD [1].

This is particularly significant in this population, especially when individuals may not always be able to articulate their own desires. While not every individual can express themselves, it is crucial at such times to take into account the words of those who came before them, families of individuals with IDD who were able to share their experiences. 

In a qualitative study based on interviews with these patients, the patients themselves described how meaningful these experiences were [1]. All the interviewed subjects unanimously enjoyed their time as 'normal' tourists and expressed a desire to travel more in the future. If they were able to enjoy, remember, and long for the travel experience, what prevents others from doing the same?

The only consistent aspect of experiences among families of individuals with IDD is their high level of inconsistency, as they significantly vary from one another in terms of cognition, adaptive functioning, financial means, available support, and the unique challenges they face on a particular excursion [2].

While medical procedures like catheterization, suctioning secretions, and providing nutrition through a gastrostomy tube have become routine for certain families, each aspect of these procedures becomes intricate when they step outside their home environment. This complexity includes the need to pack an abundance of necessary supplies, adhere to airline policies, locate suitable and hygienic settings, and be prepared for possible last-minute changes in plans [3].

For some individuals, it may be the fear or feeling of scrutiny by strangers regarding the behaviors of their relatives or loved ones [4]. Although these behaviors may not directly influence those in their immediate surroundings, the restricted yet essential expression of diverse needs or emotions through vocalizations and movements can occasionally attract uncomfortable notice. While every parent may dread a child's tantrum during an outing, for families of individuals with IDD, such outbursts can result in extreme agitation, aggression, self-harm, or sleepless nights.

Financial difficulties are a significant barrier, exacerbated by diminishing government support. This challenge is especially pronounced among families that ultimately decide that one parent must become a full-time caregiver, leading them to sacrifice their dual-income household for the well-being of their child. It is difficult to quantify the financial impact of having a child with IDD across different etiologies, but there have been several studies on conditions like Down syndrome which describe the permanent losses in earning potential and household savings [5]. In general, these financial constraints restrict spending on leisure activities, including travel beyond their local area. Moreover, a lack of awareness and inconsistent inclusivity standards within the tourism industry further discourage travel, as many destinations struggle to provide suitable accommodations for these families. 

In the end, there is no quick solution for facilitating travel for families with individuals with IDD. Still, just as the experience of travel holds many parallels to attending a doctor’s appointment, the primary care physician can play a crucial role in empowering families to make informed travel decisions. From the moment that the individual with IDD and their family express interest in travel, the physician can begin performing a pre-travel medical assessment to consider what obstacles the family might face upon traveling beyond their regular boundaries. This assessment includes considering the portability of the patient’s medications, mobility device durability, accessibility to larger quantities of medical supplies, and even helping to create a comprehensive emergency plan for the family if plans go awry.

At this point, it is also appropriate to, if possible, consult with a social worker to assist in these preparations and to consider creating a document summarizing the patient’s medical condition to help in the event of requiring medical care in their travel location of choice. There may even be a need for an entire appointment in preparation for a vacation to ensure stability of all pre-existing conditions and to also address preventative measures that may be indicated. For example, patients with severe motor and intellectual disabilities (SMIDs) are at an increased risk of deep venous thrombosis at baseline [6]. Therefore, longer travel times further increase their risk of a vascular concern and should be taken into account prior to any travel. Similarly, individuals who are nonverbal may be unable to communicate more complicated feelings such as dehydration or motion sickness. To counter this, maintaining a log of the individual’s fluid intake at home prior to vacation can ensure that families are able to continue the same or increased level of hydration while away from home. Table 1 provides travel recommendations for families with individuals with IDD.

**Table 1:** Tailored travel preparation recommendations for individuals with different developmental disabilities DD: Development disability; PCPs: Primary care physicians

DD condition	Specific and practical advice from PCPs for travel preparation
Autism spectrum disorder (ASD)	Advise on creating a travel kit with familiar items to help manage sensory issues (e.g., noise-canceling headphones and comfort objects). Recommend a pre-travel routine to gradually introduce the concept of travel. Provide a medical letter detailing the ASD diagnosis and any accommodations needed during travel (e.g., priority boarding and special seating).
Down syndrome	Ensure all routine health checks are up-to-date, focusing on cardiac and respiratory health. Discuss the importance of hygiene practices, especially if the individual is immunocompromised. Offer a list of symptoms and signs to monitor during travel that could indicate medical attention is required.
Cerebral palsy	Provide a detailed letter describing the patient’s mobility needs and equipment (e.g., wheelchair specifications and special seating requirements) for airlines and accommodations. Discuss medication management, especially for muscle spasticity, during long flights or drives. Offer advice on how to handle muscle stiffness or discomfort during travel, including stretching exercises and positioning.
Fragile X syndrome	Discuss strategies to manage anxiety in new environments, such as a familiarization routine before the trip. Review and adjust any medication for anxiety or mood disorders specifically for the travel period. Suggest carrying a detailed daily schedule to help maintain routines and reduce stress.
Fetal alcohol spectrum disorders (FASDs)	Prepare a summary of the individual’s daily routine and strategies to maintain it during travel. Offer guidance on how to handle behavioral challenges in unfamiliar settings, with an emphasis on patience and consistency. Advise on the importance of close supervision and safety, especially in crowded or new environments.

Despite thorough trip preparations and efforts to prevent potential issues, it's crucial to acknowledge that there are occasions when the individual may not fully enjoy the vacation. To prepare for this challenging but ever-present possibility, it is vital for families to invest in trip insurance. This insurance enables them to recoup a portion of their initial expenses and minimize the likelihood of harboring resentment toward the individual for circumstances often beyond their control.

By offering any number of the supportive measures listed above, the physician and other members of the medical team can facilitate an extraordinary experience for these patients and their families. Even a trip short in duration and distance from the home can be life-changing and relieve caregiver burnout. It is also crucial to acknowledge the fact that this problem stretches far beyond the individual and their primary care provider. Addressing these challenges requires collaboration between governments, the tourism industry, advocacy groups, and the community. 

Conclusions

In conclusion, the challenges faced by families of individuals with IDD in pursuing leisure travel can indeed be daunting. However, it is important to emphasize that these families should not be deprived of the joys of exploring the world as tourists. With the invaluable support of primary care providers, these families can find the means to overcome obstacles, ensuring that their loved ones can experience the wonders of travel and enjoy a fulfilling life.
